# Distinct prokaryotic gut microbiome and proviral-immune axes of pathophysiology in Sickle Cell Disease

**DOI:** 10.64898/2026.04.13.718288

**Published:** 2026-04-15

**Authors:** Zachary N. Flamholz, Jennifer De Los Santos, Karen Ireland, Janine Keenan, Jacob S. Kazmi, Aakash Mahant Mahant, Anayeli Correa, Paul S. Frenette, Betsy C. Herold, Deepa Manwani, Libusha Kelly

**Affiliations:** 1 Department of Systems and Computational Biology, Albert Einstein College of Medicine; Bronx, New York, USA; 2 Department of Pediatrics, Montefiore Health System and Albert Einstein College of Medicine; Bronx, New York, USA; 3 Department of Cell Biology, Albert Einstein College of Medicine; Bronx, New York, USA; 4 Department of Microbiology and Immunology, Albert Einstein College of Medicine; Bronx, New York, USA

## Abstract

Sickle cell disease (SCD) is a chronic, inherited condition rising across the globe. Prior studies revealed a direct link between the gut microbiome and disease micropathology via aged-like (ANs) neutrophils in mouse models. In SCD patients community-level shifts in the gut microbiome included decreases in diversity and the Firmicutes/Bacteroides (F:B) ratio, coupled to a loss of short chain fatty acid producing microbes and a shift to non-canonical butyrate production and aerobic fatty acid oxidation pathways. ANs and the proviral microbiome associate with multiple blood cytokines, while bacterial gut microbiome features largely do not. Prophages depleted of genes related to lysis, transcriptional regulation, and host takeover were enriched in SCD patient guts, pointing to domestication of these elements, and 25% of prophages were shared at high identity between study patients. In sum, we identify a viral-immune axis in SCD pathophysiology and targetable functional alterations to the gut microbiome in a heterogeneous chronic disease both affected by and effecting microbiome composition and function.

## Introduction

Sickle cell disease (**SCD**) is a chronic, inherited hematologic disorder causing significant morbidity and mortality across the globe. SCD is characterized by pathologic red blood cell hemolysis leading to complications stemming from vaso-occlusion and vascular stress, ultimately resulting in end organ damage. In a 2018 study, it was estimated 1,950 children are born with SCD in the U.S. annually, and the life expectancy of a child with SCD is 54 years compared to 76 years for the age- and race-matched U.S. population^1^. While there has been a steady increase in life expectancy for SCD patients resulting from diagnostic screening, early intervention, and better evidence-based treatment guidelines, SCD hospitalization rates have increased^2^. Pain crises, the clinical manifestation of acute vaso-occlusion, were the number one reason for SCD-related hospital admissions from 2004–2012^2^. While gene therapy will play an important role in SCD treatment, there are significant barriers to widespread utilization^3^ and with SCD births on the rise globally^4^ alternative avenues of treatment must be pursued. In addition, novel biomarkers are valuable to better stratify SCD patients for targeted early intervention and more precise treatment decisions.

The gut microbiome is a physiological system with complex interactions with SCD. It was shown in a murine model of SCD that microbial antigens transit from the gut to the bloodstream where they activate neutrophils to a disease-promoting phenotype, termed ”aged”, or ”aged-like”, neutrophils (hereafter, ”**ANs**”), and that depletion of gut microbes using broad-spectrum antibiotics decreased the AN population and improved inflammation-induced organ damage^5^. Human SCD patients were also found to have higher levels of the microbial antigen lipopolysaccharide in their blood and an increased AN percentage when compared to a control group of iron deficiency anemia patients with similar hemoglobin levels^6^. These findings led to the proposal of gut-targeting antibiotics as a treatment for SCD, and small studies in human subjects have shown antibiotics reduce the fraction of ANs and lipopolysaccharide in SCD patients^7–9^. The clinical momentum, though, has outpaced investigation of the SCD gut microbiome itself.

Gut microbiome studies that seek to identify microbial biomarkers of disease or disease severity look for abundance differences in individual taxa between case and control cohorts, as well as differences in summary measures related to community diversity. To date, there have been four studies that probed bacterial composition of the SCD gut microbiome compared to controls to determine if there are characteristic features of the SCD gut microbiome. Lim et al. did not observe differences in overall diversity for 35 SCD patients compared to a sickle trait control cohort, though one genus of Bacteroidetes had lower relative abundance in the SCD cohort^10^. Similarly, in a cohort of 32 pediatric SCD patients, Mohandas et al. did not observe a difference in alpha diversity or beta diversity compared to an immunocompromised cohort and a non-immunocompromised control cohort^11^. In contrast, Brim et al. observed patterns of abundance differences at all taxonomic levels below phylum when 14 SCD patients were compared to healthy controls^12^. Brim et al. also observed that SCD patients have a lower Firmicutes:Bacteroidetes (**F:B**) ratio, a gut microbiome community-level metric relevant to human health^13^. Finally, in a cohort of Angolan SCD pediatric patients, Delgadinho et al. did not observe a difference in alpha diversity compared to healthy siblings but did observe abundance differences at all taxonomic ranks^14^, though fewer in number and mostly different than observed by Brim et al.

These prior studies suffer from two limitations in characterizing the gut microbiome of SCD patients. First, all studies suffered from small cohort sizes, limiting their ability to detect statistical differences in microbial taxa and population metrics. Second, the studies utilized 16S sequencing for community profiling, which cannot resolve bacterial species/strain level variation, cannot identify other microbes such as viruses, and underperforms relative to metagenomic sequencing in identifying low abundance taxa^15^. Additionally, these studies found different changes in the SCD microbiome, leading to differing conclusions about its relationship with the disease. A robust evaluation of SCD patients’ gut microbiomes, together with clinical data, is necessary to determine whether there exists a relationship between the microbial community and disease pathology.

Microbiome markers have been shown to stratify treatment response in other diseases^16,17^, providing direction for the development of clinically-meaningful gut microbiome biomarkers. Additionally, gut bacteriophage populations have been recognized for their association with chronic disease^18^ and have been shown to shift lifecycle distribution at the population level in inflammatory bowel disease^19^. Interrogation of the SCD microbiome, in conjunction with studying the effect of AN levels in SCD patients, could identify novel biomarkers of disease severity in SCD. Thus, we conducted a study of blood and stool samples from patients with SCD compared with age and race matched controls. Using whole community metagenomics, we investigated the gut microbiome on multiple axes, revealing complex yet informative patterns of microbiome change. By comparing gut microbiome signatures with blood AN and cytokine levels, we begin to characterize the pathological interplay between the gut microbiome and immune system activation in SCD.

## Results

### Sickle cell disease patient cohort

Patients and controls in the study are followed at a large hospital system in the United States. Cohorts were matched for age, sex, ethnicity, and race (Supplemental Table 1). For patients, clinical history (Supplemental Table 2) and treatment history (Supplemental Table 3) were aggregated from the clinical record. Additionally, laboratory measurements were collected from the clinical record by their most recent reading (Supplemental Table 4) or assayed as part of the study (Supplemental Table 5).

### Gut microbiome changes in sickle cell disease

**Figure 1:**
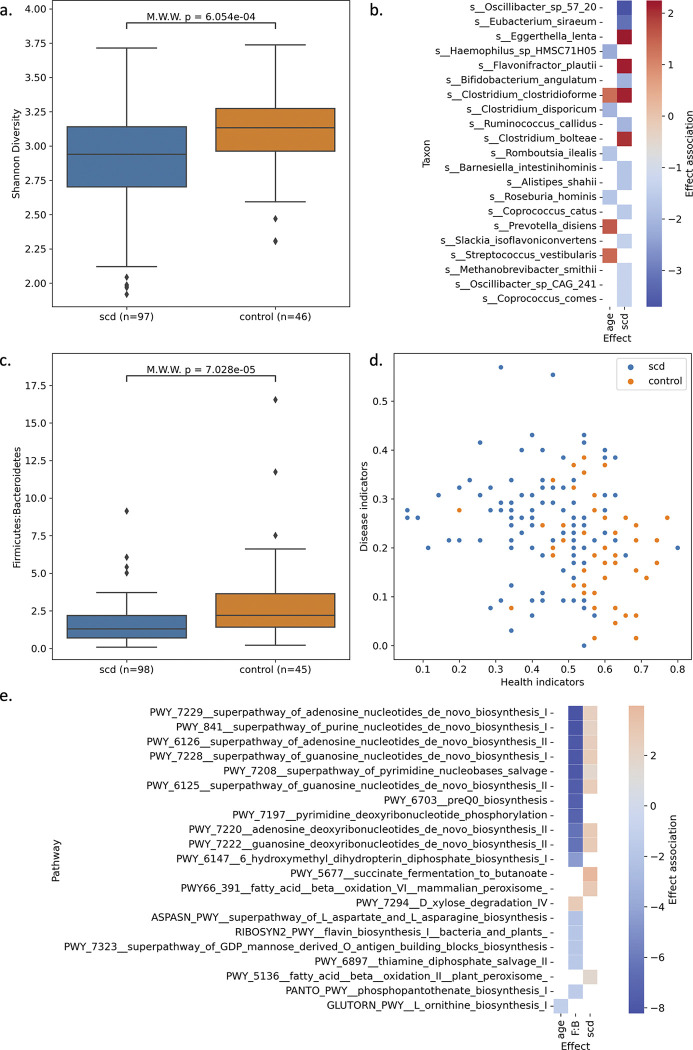
Gut microbiome community changes in SCD patients (n=98) compared to healthy controls (n=46). Sample whole community sequencing was profiled using the MetaPhlAn taxa marker database^20^. (a) Distribution of sample alpha diversity measured as Shannon diversity for patients and controls. (b) A generalized linear model was used to determine the effect size and association of bacteria taxa abundance with sample metadata including SCD status, age, race, ethnicity, and gender, all modeled as fixed effects. Taxa with q-value < 0.05 for any effect are shown. (c) Distribution of Firmicutes to Bacteroidetes (F:B) ratio in the gut microbiome of patients and controls. (d) Scatter plot of health and disease indicator scores per sample. (e) Sample whole community sequencing was profiled using the HUMAnN pathway marker database^20^. A generalized linear model was used to determine the effect size and association of pathway abundance with sample metadata including SCD status, age, race, ethnicity, and gender, all modeled as fixed effects. Additionally, the F:B was also included as fixed effect. Pathways with q-value < 0.05 for any effect are shown. In a and c, samples with values +/− 3 s.d. from mean of all samples were removed. M.W.W.- Mann-Whitney-Wilcoxon test.

To study the SCD gut microbiome, we sequenced fecal samples from patients and controls. Sample sequencing reads were profiled for microbial taxa using a library of taxa-specific marker genes^20^. To determine whether there is a shift in the SCD gut microbiome at the community level, we used Shannon diversity to compare the species richness between patients and controls and found that SCD patients have less diverse gut microbiomes (Figure 1a). To identify microbes positively or negatively associated with SCD, we modeled taxa abundance as a function of age, sex, ethnicity, race, and SCD status using a multivariable generalized linear model (Supplemental Table 1). Overall, 25 taxa are significantly associated with SCD and 6 taxa are associated with age (Figure 1b). We noted that SCD samples had higher abundances of the phylum Bacteroidetes (**B**) and lower abundances of the phylum Firmicutes (**F**), the two taxonomic groups that make up the composite metric F:B ratio, and observe a reduction in the ratio in our study (Figure 1c), as had been observed previously^12^. Looking at human gut microbiome indicator species identified in a meta-analysis of human microbiome studies^21^, we observed a decrease in health indicators (p=5.6e-9; Mann–Whitney–Wilcoxon test) and increase in disease indicators (p=4.4e-3; Mann–Whitney–Wilcoxon test) in SCD patients compared to controls (Figure 1d), pointing to changes in the SCD gut microbiome that are consistent with changes observed in other human diseases.

We also performed a functional characterization of samples using gene-based pathway annotation^20^. We again modeled abundance with the same fixed effects but included an additional effect, the F:B ratio, to both capture community-level changes and identify SCD-specific changes that are independent of the high-level shift (Figure 1e). High F:B ratio had exclusively negative associations that were significant, with the most significant associations with pathways related to nucleotide biosynthesis. SCD status was positively associated with three pathways related to butyrate fermentation, fatty acid oxidation, and vitamin B6 biosynthesis, and negatively associated with a pathway related to anaerobic metabolism. The small number of pathways robustly associated with SCD in our heterogeneous patient population point to specific functional changes that shed light on conserved contributions of the gut microbiome to SCD pathology.

### Gut microbiome markers and known SCD biomarkers

We investigated whether gut microbiome changes in SCD are related to the AN population by asking whether microbiome markers correlate with the fraction of ANs of the total neutrophil population in a patient’s blood (Supplemental Figure 1). For this analysis, we utilized a subset of the SCD cohort whose blood was assayed (n=57). Controls were not assayed for ANs and are excluded from the following analysis. We evaluated four groups of markers: (1) species positively associated with SCD- *E. lenta*, *F. plautii*, and *C. bolteae*; (2) species negatively associated with SCD- *O. sp. 57 20*, *E. siraeum*, textitA. shahii, *B. intestinihominis*, *R. callidus*, *B. angulatum*, *C. catus*, *S. isoflavoniconvertens*, *M. smithii*, *O. sp CAG 241*, and *C. comes*; (3) functions significantly different between patients and controls- succinate fermentation to butanoate, fatty acid beta oxidation VI mammalian peroxisome, and fatty acid beta oxidation II; and (4) system-level summary metrics shown to be differential between patients and controls- Shannon diversity, F:B ratio, health indicators, and disease indicators. A single marker, the bacterial species *A. shahii*, significantly correlated positively with the AN percent (*ρ* = 0.26, p=0.048).

Given the substantial changes in the gut microbiome of SCD patients, we next investigated whether gut microbiome features are associated with SCD clinical and molecular measures of disease severity. We utilized a molecular blood panel to profile SCD patients for levels of cytokines and chemokines (Supplemental Table 5). Additionally, we included a number of hemolysis-related clinical measurements (Supplemental Table 4).

**Figure 2:**
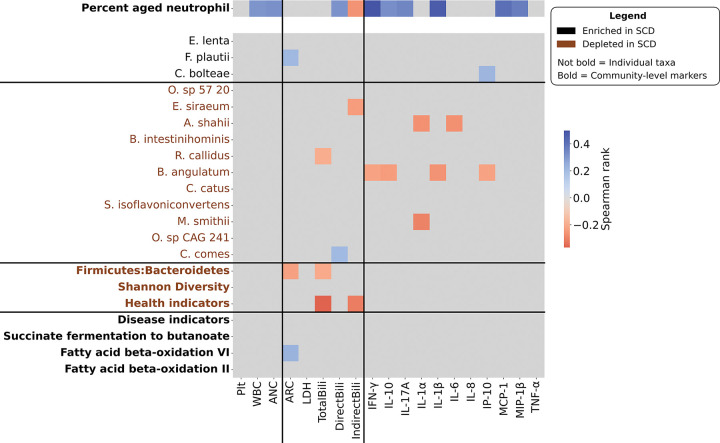
Aged neutrophil percentage (n=57) and gut microbiome feature (n=98) correlations with clinical measures and blood molecular immune markers. Correlations were measured with Spearman *ρ* and significance was measured using permutation testing. Value of correlation is colored for strength of correlation where p < 0.05, otherwise comparison cell is gray. For the number of samples assayed for each clinical and molecular measure, see Supplemental Tables 4–5.

We first evaluated the AN fraction given its known role in SCD pathology. Neutrophil activation assay measures had significant correlations with white blood cell measures collected from the health record (Supplemental Figure 2), providing confidence in comparing AN data with clinical data. ANs were significantly correlated with four inflammatory cytokines: interferon gamma, interleukin-1*β*, interleukin-10, and interleukin-17A, and two chemokines: monocyte chemoattractant protein-1 and macrophage inflammatory protein-1B (Figure 2, top). In contrast, most of the gut microbiome metrics and all of the community metrics did not correlate with inflammatory markers (Figure 2, bottom). Exceptions to this pattern are *A. shahii* and *B. angulatum*, both negatively associated with SCD and inversely correlated with multiple marker levels. Gut microbiome features did have significant correlations with clinical disease markers, including absolute reticulocyte count (**ARC**), total bilirubin, and indirect bilirubin.

### Gut microbiome viruses

**Figure 3:**
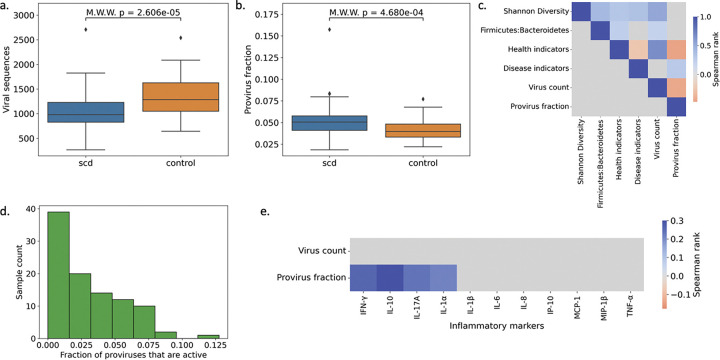
Gut microbiome viral population is altered in SCD. Sample whole community sequencing was assembled and viral sequences were identified using marker genes and sequence features. (a-b) The number of viral sequences (a) and the fraction of provirus sequences (b) between patients and controls. (c) Comparison of viral population and bacterial population community-level metrics. (d) Histogram of active provirus fraction for SCD samples. (e) Correlation of viral community-level metrics with clinical and molecular markers as in 3b and 3c. Value of correlations is shown for comparisons with p < 0.05 otherwise comparison cell is gray. M.W.W = Mann-Whitney-Wilcoxon test.

Having observed limited correlation between bacterial gut microbiome markers and ANs, we interrogated the viral population in metagenomic samples. Assembled metagenomes were profiled for viral sequences using a hybrid approach that combines viral marker genes and sequence features to distinguish chromosomal, plasmid, and viral sequences^22^. Overall, there was a decrease in the number of viral sequences in SCD compared to controls (Figure 3a), likely reflecting the loss in diversity observed in SCD samples given its correlation with sample Shannon diversity and health indicators (p=2.6e-5; Mann–Whitney–Wilcoxon test) (Figure 3c). Proviruses are lysogenic virus sequences integrated into host genomes and can be labeled in assembled viral genomes by identifying sequence features such as viral integrase proteins and integration sites^23^. Proviruses constitute a small fraction of the viral sequences present in samples, yet there was a significant enrichment of provirus sequences in SCD compared to controls (p=4.7e-4; Mann–Whitney–Wilcoxon test) (Figure 3b), which was robust to provirus or lysogenic viral sequence calling method (Supplemental Figure 2). While the provirus fraction was not correlated with bacterial community-level metrics Shannon diversity and F:B, it was inversely correlated with health indicators and positively correlated with disease indicators (Figure 3c), providing evidence that provirus enrichment mirrors changes in the bacterial species present in the sample.

From assembled provirus sequences it is not possible to determine whether a provirus is active, meaning replicating independently of its host via a lytic lifecycle, or inactive. Using a method developed previously^24^, we analyzed the sequencing read distribution of provirus sequences and their flanking host sequences to determine if there was increased read coverage of proviruses, which would indicate independent replication. Across SCD samples, >90% of proviruses were inactive (Figure 3d), and the active provirus fraction distribution in SCD was not different from controls (p=3.208e-1; Mann–Whitney–Wilcoxon test). Finally, we performed the same correlation experiment for viral-based metrics as we performed for bacterial markers. While the provirus fraction did not significantly correlate with AN fraction (*ρ* = 0.02, p=0.91), the provirus fraction had a number of statistically significant positive correlations with molecular cytokines (Figure 3e), which was not true of non-integrated temperate phages (Supplemental Figure 4).

### Integrated viruses

After finding that the provirus fraction in the gut microbiome correlated with the level of multiple inflammatory cytokines in SCD patients, we decided to further investigate the provirus sequences. A total of 4,962 proviruses were identified across the SCD patient gut microbiomes. Provirus lengths had a bimodal distribution with a median size of 23,775 base pairs (Supplemental Figure 5). All proviruses with predicted taxonomy (99.5%) were bacteriophages of the class Caudoviricetes (99.3%), meaning the proviral sequences are prophages.

**Figure 4:**
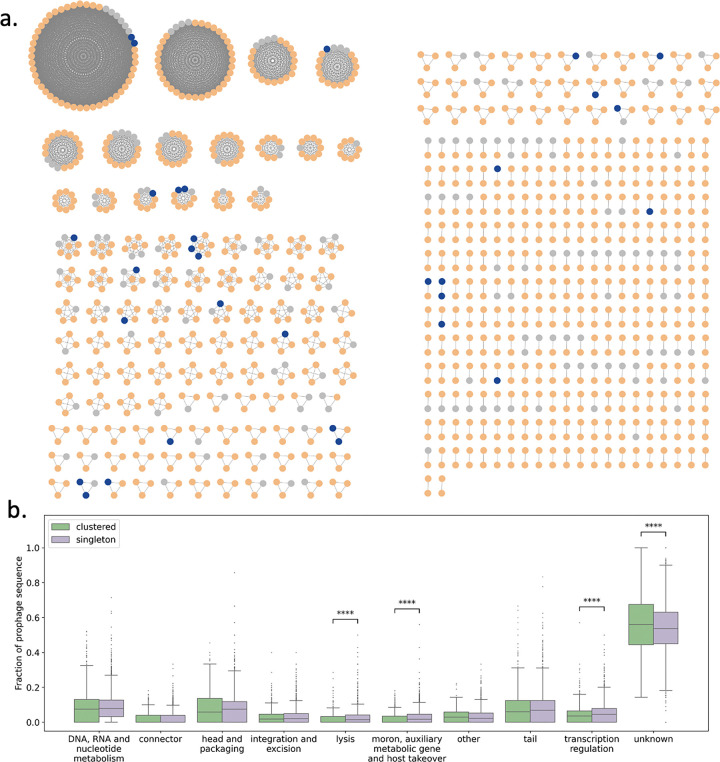
Highly conserved prophage sequences are shared by gut microbiomes of SCD patients. (a) Patient microbiomes sharing a highly conserved prophage sequence visualized as fully connected networks. Conserved prophages had 99% sequence identity over at least 70% of bidirectional sequence coverage. Node color indicates predicted prophage activity: active (blue), dormant (orange), and undetermined (gray). (b) Prophage sequence distribution of high-level viral protein functions compared between clustered (n=1,249) and singleton (n=3,713) prophages. Significance tested with a Mann-Whitney-Wilcoxon test: **** = p < 0.0001.

Because the vast majority of phages were categorized as inactive in host bacterial genomes, we wondered whether these sequences would be similar between patients. We clustered all prophage sequences at 99% identity over at least 70% bi-directional coverage and found 395 clusters of sequences with median size of 2 (Figure 4a). In total, 1,249 (25%) sequences had a nearly identical sequence in another sample in the dataset. These clustered prophages were homologous to sequences in common gut commensals, however Bacteroides stood out with 113 clusters matching species in the genus (Supplemental Figure 6). The decreased F:B ratio in our patient population may also represent an increase in Bacteroides strains carrying these conserved prophages, although at the community level, the prophage fraction did not inversely correlate with the F:B ratio as would be expected if it were such strains driving the community shift. To determine whether clustered prophages differed from singletons, we compared the high-level functional content of sequences in each group (Figure 4b). Interestingly, clustered prophage sequences had lower fractions of genes with functions related to lysis, transcriptional regulation, and host takeover and a higher fraction of genes with unknown function compared to non-clustered prophages. A reduction in proteins that function in host lysis was also observed in active prophages compared to dormant prophages (Supplemental Figure 7).

## Discussion

The primary goal of our study was to ask whether there exist consistent, significant interactions between the gut microbiome and disease pathology in SCD. Such interactions, when observed across heterogeneous patients with varying clinical histories and burdens of disease, could lead to novel treatment modalities and diagnostics to improve patient care and monitoring in this disease of increasing importance around the world. With orthogonal computational and experimental approaches, we can begin to build a model of the interactions between gut microbiome features, immune system activity, and pathology in SCD.

**Figure 5:**
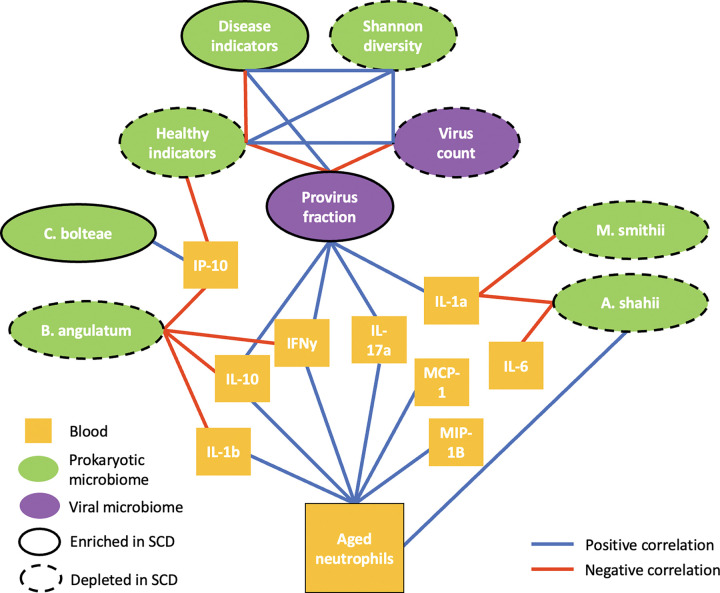
A model of interactions between the gut microbiome and markers of inflammation in the blood of SCD patients. The gut microbiome can influence and is influenced by the immune system. Using multiple data modalities, we begin to understand the complex interaction of gut microbiome changes and immune system activation in the pathology of SCD.

Our study reveals a two-tiered dialogue between the gut ecosystem and systemic inflammation in SCD (Figure 5). Shotgun metagenomics of 98 patients showed a disease-wide contraction of bacterial diversity, a drop in the F:B ratio, and characteristic changes in pan-disease indicator taxa. These shifts parallel patterns seen across disparate chronic illnesses^21,25^, suggesting that SCD provokes a generic disease signature at the microbiome community level.

Neutrophil biology and the microbiome appear to occupy distinct, complementary niches. The AN subset correlated with an overlapping cytokine panel but not with bacterial community metrics. Conversely, bacterial *α*-diversity and indicator taxa associated with indirect bilirubin and reticulocyte count—clinical proxies for hemolysis, yet show little relationship to the immune milieu. This dissociation implies that neutrophil activation and microbial changes represent parallel arms of SCD pathophysiology, each informative for a different disease facet.

Beyond bacteria, dormant prophages emerge as a previously unrecognized axis of SCD biology. We detect a 1.7-fold increase in the prophage fraction, yet >90% of these elements are predicted to be replicatively silent. Strikingly, the proportion of prophages, but not total phage population or lytic phage fraction, tracks with IL-1*α*, IL-10, IL-17A and IFN-*γ* levels. Immune signaling molecules are known to be elevated at baseline in SCD patients^26–28^, and 3/4 cytokines correlated with prophage level are observed to be lower in patients in vaso-occlusive crisis compared to SCD patients not in vaso-occlusive crisis, including IL-10, IL-17A, IFN*γ* ^26^. Interestingly, the gut microbiome in SCD patients harbors prophage sequences that are highly conserved between unrelated individuals, with one such sequence present in over 50% of metagenomes. These conserved, shared prophages were an unexpected feature of SCD patient microbiomes, and stand in contrast to studies of lytic phages, that found non-integrating bacteriophage populations are highly individual-specific^29^. We found that clustered sequences had a higher fraction of genes coding for proteins involved in host takeover, gene expression manipulation, and lysis. We speculate that lysogens bearing ‘domesticated’ prophages maintain a survival advantage in the setting of a disease-associated gut microbiome. Enrichment of lysogens may modulate host immunity by altering bacterial surface antigens or metabolite flux. Experimental induction assays and longitudinal sampling will be needed to test whether prophage excision precedes cytokine spikes or merely registers host stress.

Functional signature investigation of the gut microbiome, enabled by metagenomics, can have therapeutic implications^30^. Among the functional module changes observed in SCD, three merit particular attention: (i) enrichment of nucleotide biosynthesis pathways, (ii) succinate to butanoate fermentation, a non-canonical route for microbial butyrate generation, and (iii) fatty-acid *β*-oxidation pathways, which typically require molecular oxygen and are not common in the anaerobic gut environment. The communal shift toward a low F:B ratio was tightly coupled to an expansion of nucleotide-biosynthesis modules, offering a narrow-spectrum antimicrobial target set that could re-balance the ecosystem more precisely than broad antibiotics trialed to date^7–9^. Looking at functional modules enriched in SCD independent of community shift, we see the succinate to butanoate butyrate production pathway; a departure from the primary gut butyrate acetyl-CoA pathway^31^. Butyrate is the primary energy source for gut epithelial cells and has been shown to be anti-inflammatory and supportive of epithelial-barrier integrity^32^. This shift in butyrate production pathway in the SCD gut may reflect the loss of members of taxonomic groups that produce butyrate via complex carbohydrate fermentation, such as C. catus, C. comes and E. siraeum^31,33^. Conversely, elevated *β*-oxidation genes suggest greater flux through short- and medium-chain fatty-acid catabolism, a shift that might deplete beneficial metabolites and warrants metabolic follow-up. Notably, the SCD enriched *β*-oxidation pathways II and IV are mitochondrial and peroxisomal, and therefore generally aerobic, pointing to the availability of molecular oxygen in the gut, a hallmark of gut barrier disruption^34,35^.

There are a number of recognized limitations for our study. First, while we collectively grouped SCD patients into a single cohort, the disease can vary in its presentation and clinical course, which may affect the gut microbiome. Our cross-sectional design precludes causal inference and we acknowledge that as a chronic disease, SCD presentation can be highly variable. Second, flow-cytometry assays for neutrophil activation markers were performed within 8 hours of venipuncture, and while we have correlative evidence that our results reflect accurate measures, we did not formally benchmark marker stability to this time window. Next, prophage activity was inferred from coverage ratios, as is common in the field, but with this method asynchronous induction events could evade detection. Finally, findings derive from a single U.S. center and may not generalize to global SCD populations with different diets or genotypes.

In summary, we propose that the gut microbiome bacterial shift mirrors hemolytic burden while ANs and dormant prophages mirror immunological activation tone. Longitudinal and interventional studies, particularly those integrating metatranscriptomics and targeted bacteriotherapy, are now warranted to unravel causality and translate these markers into clinical tools.

## Methods

### Study design and subjects

The study is a single-center outpatient cohort study of SCD patients and age- and race-matched controls. Study participants were recruited from the Children’s Hospital at Montefiore and Montefiore Medical Center (IRB NUMBER: 2018–9080). Patients were enrolled with the following inclusion criteria: 1) diagnosis of sickle cell anemia (sickle cell disease SS genotype or sickle beta zero thalassemia, S*β*^0^-thal), or sickle cell trait in association with hereditary persistence of fetal hemoglobin (S-HPFH); 2) age ≥ 4; and 3) currently in a usual state of health. Exclusion criteria included: 1) malignancy; 2) inflammatory bowel disease or other gastrointestinal disorder; 3) immunocompromised due to additional underlying disease or immunosuppressive medication; 4) history of hematopoietic stem cell transplant; 5) history of C. difficile infection in the preceding 2 months; 6) receipt of chemotherapy in the preceding 2 months; 7) receipt of systemic antibiotic other than Pen VK in the preceding 2 months; 8) Hospitalization within the preceding 2 weeks; 9) intercurrent febrile illness or sickle cell related pain episode requiring opioids within the preceding 2 weeks. Controls were included with age ≥ 4 and excluded with the same criteria in addition to any systemic antibiotic use in the preceding 2 months.

In total, 101 SCD patients and 66 controls were enrolled in the study from 2018–2020. Of these participants, 98 SCD patients and 46 controls provided stool samples. One patient sample did not have enough material for metagenomic sequencing. Fecal samples were collected by study participants into DNA/RNA Shield Fecal Collection tube (Zymogen Research) and samples were split into 6 tubes and stored at −80°C. Blood was also drawn from study participants around the time of fecal collection.

### Clinical and molecular data

Clinical data was collected for SCD patients based on chart review. Information included: (i) demographics- age (years), sex, ethnicity, race, hemoglobin genotype (SS, S*β*^0^, SS with high F, AS, AA, where ‘S’ indicates a sickle cell allele, ‘*β*^0^’ indicates a thalassemia allele coding for no wild type hemoglobin, ‘F’ indicates a fetal hemoglobin allele, and ‘A’ indicates a wild-type allele); (ii) patient history- hydroxyurea (on/off), folic acid (on/off), glutamine (on/off), stroke (yes/no), asthma (yes/no), allergies (yes/no), bacteremia (yes/no), silent infarct (yes/no), history of meningitis, osteomyelitis, or urinary tract infection (yes/no), number of acute chest syndrome past year, number of pain admissions past year, number of 30 day readmissions past year, number of emergency room visits past year, number of transfusions in the past year, number of exchanged transfusions in the past year; and (iii) clinical measurements- weight (kg), body mass index (BMI), urine microalbumin (MA/Creatinine; mg/gm), lactate dehydrogenase (LDH; U/L), platelet (PLT; k/uL), white blood cells (WBC; k/uL), absolute neutrophil count (ANC; k/uL), hemoglobin (Hb; g/dL), sickle hemoglobin (HbS; %), fetal hemoglobin (HbF; %), wild type hemoglobin (HbA; %), absolute reticulocyte count (ARC; k/uL), total bilirubin (mg/dL), direct bilirubin (mg/dL), indirect bilirubin (mg/dL), alanine aminotransferase (ALT; U/L), Creatinine (mg/dL), iron studies: iron level (ug/dL), transferrin (ug/dL), saturation (%), total iron binding capacity (TIBC; ug/dL), ferritin (ng/dL). All yearly measures were annualized to 1 year if patient records are < 1 year in duration. Acute care in the past year is the sum of pain admissions and emergency department visits.

Venous blood in Acid Citrate Dextrose was collected from study participants. Inflammatory cytokine and chemokine levels were measured from serum using the MILLIPLEX Human Cytokine/Chemokine/Growth Factor Panel A kit (MilliporeSigma, HCYTA-60K). Luminex assay data were acquired on a Luminex MAGPIX and analyzed with the MILLIPLEX Analyst program (MilliporeSigma). The following cytokines and chemokines were profiled: G-CSF (pg/mL), IFN*γ* (pg/mL), IL-10 (pg/mL), IL-17A (pg/mL), IL-1*α* (pg/mL), IL-1*beta* (pg/mL), IL-6 (pg/mL), IL-8 (pg/mL), IP-10 (pg/mL), MCP-1 (pg/mL), MIP-1*β* (pg/mL), TNF-*α* (pg/mL). Cytokines and chemokines with < 50% of samples assayed at the lower limit of detection were excluded from further analysis.

### Neutrophil activation biomarker assessment

Blood was also evaluated by flow cytometry for neutrophil adhesion and activation markers. Flow cytometry was performed using LSRII equipped with FACS Diva 8.0.1 software (BD Biosciences) and analyzed with FlowJo software (Tree Star). Neutrophils were identified by forward and side scatter characteristics combined with CD16b expression, and the AN subset evaluated by CD62L^lo^ CXCR4^hi^ expression within the neutrophil population. Whole blood from the same sample used for flow cytometry assays was diluted 1:10 in PBS for complete blood count with differential counts on ADIVA 120 (Siemens Healthcare Diagnostics). Total WBC and absolute neutrophil counts along with percent AN from flow cytometry were used to calculate the absolute aged neutrophil count. Samples were excluded if analyses were not performed within 8 hours of blood draw.

### DNA extraction and sequencing

Sample preparation and sequencing was done by the Molecular Microbiology Facility of the Integrated Genomics Operation at Memorial Sloan Kettering Cancer Center. DNA was extracted from samples using a custom phenol chloroform extraction optimized for fungal and bacterial isolation. Metagenomic sequencing was performed using next generation sequencing with the Illumina HiSeq platform using 2×150bp paired end reads. For some samples, multiple sequencing runs were performed to achieve targeted sequencing depth. Base calling was performed by platform software resulting in paired FASTQ files for each sample. Samples with multiple runs were concatenated to single paired files.

### Metagenomic sequence profiling

For bacterial taxa and function analysis, the bioBakery 3 suite of metagenomic sequence tools was used^20^. First, raw reads were fist processed for quality and removal of human contamination using kneaddata (v0.10.0) with the following parameters -t 8 -p 12 –cat-final-output^20^. Bacterial taxa were profiled with MetaPhlAn3 (v3.0) with default parameters and –no map –nproc 12 and utilized the mpa_v30_CHOCOPhlAn_201901 clade-specific marker gene database^20^. MetaPhlAn output for all samples were collapsed to a single table using the merge_metaphlan_tables.py utility script^20^. Function pathways were profiled with HUMAnN3 (v3.7)^20^. Paired read files were merged to a single table and HUMAnN was run with default parameters and –threads 96^20^ and database versions mpa_vOct22_CHOCOPhlAnSGB_202212 and uniref90_201901b_full.dmnd. For comparison between samples, pathway profiles were normalized for read sequence depth using the script humann_renorm_table to copies per million with parameters –units cpm –update-snames; stratified profiles were split using the humann_split_stratified_table script; and merged to a single table using the humann_join_tables script^20^.

For viral profiling, raw sequences were processed with the nextflow nf-core/mag (v2.4.0) metagenomics pipeline^36,37^ for quality and contamination removal, assembly with MEGAHIT^38^, and gene calling with prodigal^39^. The pipeline was run with -profile singularity and parameters –skip_binning –skip_spades. Viral sequences were profiled from MEGAHIT assemblies using geNomad (v1.7.1) with the end-to-end command and default parameters^22^ and using VIBRANT (v1.2.1) using the VIBRANT_run.py script with -f nucl -t 8 -no_plot parameters^40^. Both methods predict provirus sequences but only VIBRANT predicts lytic vs. lysogenic bacteriophage life cycle. To calculate the non-integrated lysogenic virus count in the VIBRANT data, the count of provirus sequences was subtracted from the count of lysogenic viruses per sample. PropagAte (v1.1.0) with default parameters was used to determine the activity of provirus sequences^24^ from raw sequence reads, MEGAHIT assemblies, and provirus coordinates called by both geNomad and VIBRANT. All fraction metrics were calculated by dividing the count of interest by the total number of viruses called by the respective method (e.g. the provirus fraction for geNomad is the count of proviruses divided by the total count of viruses per sample).

### SCD metagenome analysis

Multiple metrics were calculated for the bacterial population in each sample using its MetaPhlAn profile. Shannon diversity was calculated using the MetaPhlAn utility script calculate diversity.R script with parameters -d alpha -m shannon -s s_. The F:B ration was calculated by dividing the abundance of ‘t_Firmicutes’ by the abundance of ‘t_Bacteroidetes. Health and disease associated taxa were collected from a gut microbiome meta-analysis^21^, and the number of taxa in each set was counted based on the presence of the taxa at any abundance in the sample, as performed in^21^.

To determine bacterial taxa and pathways differentially abundant in SCD patient samples compared to controls, we utilized a multivariate generalized linear model approach to model profiles as log-linear with MaAsLin 2 (R; v1.8)^41^. For taxa, MaAslin2 was run with min_prevalence = 0.1 and min_abundance = 0.0001. For pathways, MaAslin2 was run with min_prevalence = 0.1 and normalization = ‘NONE’. We included a number demographic variables as fixed effects, modeling

taxa∼scd+age+race+ethnicity+sex

pathways∼scd+age+race+ethnicity+sex+F:B

where scd is an indicator variable for the disease status of a sample and abundance refers to either taxa or pathways. Effect heat maps were generated from MaAslin2 significant result tables, and significant effects were reported for q<0.05.

### Prophages investigation

Prophage sequences identified using geNomad were clustered at 99% sequence identity over >= 70% of bidirectional sequence coverage using mmseqs2 (v14.7e284)^42^ with parameters –cluster-mode 0 –cov-mode 0 -c 0.7 –min-seq-id 0.99. Clusters were converted to fully connected networks using the python package networkx (v3.1)^43^ and visualized using cytoscape (v3.10.0)^44^. VPF-PLM^45^ was used for functional annotation of prophage sequences. Clustered prophage homology to bacterial species sequences was done using blastn (v2.16.0+) and the core_nt database with parameters evalue 1e-10 and perc_identity 90. The best hit bacterial species was assigned to each cluster where such a hit was identified.

### Statistical analyses and visualizations

Mann–Whitney–Wilcoxon was used to compare distribution means between SCD patients and controls for Shannon diversity, F:B ratio, healthy indicator fraction, and disease indicator fraction. Values were removed in +/− 3 standard deviations from mean of all study samples. Spearman’s *ρ* was used for all tested correlations with the exception of flow cytometry and electronic health record white blood cell measures which was tested with Pearson correlation coefficient. Significance of a correlation was measured using a permutation test with a two-sided alternative hypothesis. Mann–Whitney–Wilcoxon test was used for all tested association of categorical variables test with a two-sided alternative hypothesis. All methods were implemented with scipy stats package^46^: spearmanr, pearsonr, and mannwhitneyu with default parameters and permutation_test with alternative=‘two-sided’, permutation_type=‘pairings’. Significance was assigned for p < 0.05 for all comparisons. Visualizations were produced using python packages seaborn^47^ and statannotations^48^. Pandas^49^ and numpy^50^ python packages were used for analysis.

## Supplementary Material

Supplement 1

## Data Availability

The dataset ”Sickle cell disease patient gut microbiome study” is available in the repository NCBI and can be accessed via the following accession ID: PRJNA1320713. Patient level clinical, neutrophil, and immune data can be found in the Supplementary Material file.

## References

[1] Agodoa I. Societal Costs of Sickle Cell Disease in the United States. Blood, 132(Supplement 1):4706–4706, 11 2018.

[2] Ho A. T. N. Trends in hospitalizations for sickle cell disease related-complications in usa 2004 – 2012. Journal of Hematology, 8(1), 2019. 10.14740/jh475 PMC7153675 32300435

[3] Tessema F. A. High-priced sickle cell gene therapies threaten to exacerbate us health disparities and establish new pricing precedents for molecular medicine. Journal of Law, Medicine and Ethics, 50(2):380–384, 2022. 10.1017/jme.2022.66 35894560

[4] Thomson A. M. Global, regional, and national prevalence and mortality burden of sickle cell disease, 2000–2021: a systematic analysis from the global burden of disease study 2021. The Lancet Haematology, 10(8):e585–e599, Aug 2023. 37331373 10.1016/S2352-3026(23)00118-7 PMC10390339

[5] Zhang D. Neutrophil ageing is regulated by the microbiome. Nature, 525(7570):528–532, 2015. 26374999 10.1038/nature15367 PMC4712631

[6] Dutta D. Intestinal injury and gut permeability in sickle cell disease. Journal of Translational Medicine, 17(1):183, 2019. 31146745 10.1186/s12967-019-1938-8 PMC6543649

[7] Lim S. H. Rifaximin for sickle cell disease. American Journal of Hematology, 94(12):E325–E328, 2019. 31512269 10.1002/ajh.25637

[8] Dutta D. Effects of rifaximin on circulating aged neutrophils in sickle cell disease. American Journal of Hematology, 94(6):E175–E176, 2019. 30900765 10.1002/ajh.25467

[9] Dutta D. and Lim S. H.. Effects of Rifaximin on Intestinal Pathophysiologic Changes Associated with Sickle Cell Disease (SCD). Blood, 134(Supplement 1):2282–2282, 11 2019.

[10] Lim S. H. Intestinal 1microbiome analysis revealed dysbiosis in sickle cell disease. American Journal of Hematology, 93(4):E91–E93, 2018. 29274089 10.1002/ajh.25019

[11] Mohandas S. Differences in gut microbiome in hospitalized immunocompetent vs. immunocompromised children, including those with sickle cell disease. Frontiers in Pediatrics, 8, 2020. 10.3389/fped.2020.583446 PMC7690629 33282798

[12] Brim H. The gut microbiome in sickle cell disease: Characterization and potential implications. PLOS ONE, 16(8):1–15, 08 2021. 10.1371/journal.pone.0255956 PMC8386827 34432825

[13] Ley R. E. Human gut microbes associated with obesity. Nature, 444(7122):1022–1023, Dec 2006. 17183309 10.1038/4441022a

[14] Delgadinho M. Microbial gut evaluation in an angolan paediatric population with sickle cell disease. Journal of Cellular and Molecular Medicine, 26(21):5360–5368, 2022. 36168945 10.1111/jcmm.17402 PMC9639033

[15] Durazzi F. Comparison between 16S rRNA and shotgun sequencing data for the taxonomic characterization of the gut microbiota. Scientific Reports, 11(1):3030, 2021. 33542369 10.1038/s41598-021-82726-y PMC7862389

[16] Gu Y. Analyses of gut microbiota and plasma bile acids enable stratification of patients for antidiabetic treatment. Nature Communications, 8(1):1785, Nov 2017. 10.1038/s41467-017-01682-2 PMC5702614 29176714

[17] Muscolino P. Potential predictive role of gut microbiota to immunotherapy in hcc patients: a brief review. Frontiers in Oncology, 13, 2023. 10.3389/fonc.2023.1247614 PMC10486017 37692859

[18] Tisza M. J. and Buck C. B.. A catalog of tens of thousands of viruses from human metagenomes reveals hidden associations with chronic diseases. Proceedings of the National Academy of Sciences, 118(23):e2023202118, 2021. 10.1073/pnas.2023202118 PMC8201803 34083435

[19] Clooney A. G. Whole-virome analysis sheds light on viral dark matter in inflammatory bowel disease. Cell Host & Microbe, 26(6):764–778.e5, 2019. 31757768 10.1016/j.chom.2019.10.009

[20] Beghini F. Integrating taxonomic, functional, and strain-level profiling of diverse microbial communities with biobakery 3. eLife, 10:e65088, may 2021. 33944776 10.7554/eLife.65088 PMC8096432

[21] Khan S. and Kelly L.. Oral microbes are a signature of disease in the gut. 2023.

[22] A. P. Camargo Identification of mobile genetic elements with genomad. Nature Biotechnology, Sep 2023. 10.1038/s41587-023-01953-y PMC11324519 37735266

[23] Howard-Varona C. Lysogeny in nature: mechanisms, impact and ecology of temperate phages. The ISME Journal, 11(7):1511–1520, Jul 2017. 28291233 10.1038/ismej.2017.16 PMC5520141

[24] Kieft K. Deciphering active prophages from metagenomes. mSystems, 0(0):e00084–22, 2022. 10.1128/msystems.00084-22 PMC9040807 35323045

[25] Duvallet C. Meta-analysis of gut microbiome studies identifies disease-specific and shared responses. Nature Communications, 8(1):1784, Dec 2017. 10.1038/s41467-017-01973-8 PMC5716994 29209090

[26] Silva-Junior A. L. Immunological hallmarks of inflammatory status in vaso-occlusive crisis of sickle cell anemia patients. Frontiers in Immunology, 12:559925, 3 2021. 33776989 10.3389/fimmu.2021.559925 PMC7990896

[27] Garcia N. P. Sickle cell anemia patients display an intricate cellular and serum biomarker network highlighted by tcd4+cd69+ lymphocytes, il-17/mip-1¡i¿*β*¡/i¿, il-12/vegf, and il-10/ip-10 axis. Journal of Immunology Research, 2020:4585704, Jan 2020. 32411797 10.1155/2020/4585704 PMC7199620

[28] Gomes I. C. P. Levels of inflammatory markers are differentially expressed in sickle cell anemia and sickle cell trait. eJHaem, 4(3):705–709, 2023. 37601842 10.1002/jha2.712 PMC10435695

[29] Stockdale S. R. Interpersonal variability of the human gut virome confounds disease signal detection in ibd. Communications Biology, 6(1):221, Feb 2023. 36841913 10.1038/s42003-023-04592-w PMC9968284

[30] Wang Z. Non-lethal inhibition of gut microbial trimethylamine production for the treatment of atherosclerosis. Cell, 163(7):1585–1595, 2015. 26687352 10.1016/j.cell.2015.11.055 PMC4871610

[31] Vital M. Revealing the bacterial butyrate synthesis pathways by analyzing (meta)genomic data. mBio, 5(2):10.1128/mbio.00889-14, 2014. PMC3994512 24757212

[32] Zheng L. Microbial-derived butyrate promotes epithelial barrier function through il-10 receptor–dependent repression of claudin-2. The Journal of Immunology, 199(8):2976–2984, 10 2017. 28893958 10.4049/jimmunol.1700105 PMC5636678

[33] Holdeman L. V. and Moore W. E. C.. New genus, coprococcus, twelve new species, and emended descriptions of four previously described species of bacteria from human feces. International Journal of Systematic and Evolutionary Microbiology, 24(2):260–277, 1974.

[34] Zong W. Disruption of intestinal oxygen balance in acute colitis alters the gut microbiome. Gut Microbes, 16(1):2361493, 2024. 38958039 10.1080/19490976.2024.2361493 PMC11225921

[35] Ramakrishnan S. K. and Shah Y. M.. Role of intestinal hif-2*α* in health and disease. Annual Review of Physiology, 78:301–325, 2016. 10.1146/annurev-physiol-021115-105202 PMC4809193 26667076

[36] Krakau S. nf-core/mag: a best-practice pipeline for metagenome hybrid assembly and binning. NAR Genomics and Bioinformatics, 4(1):lqac007, 02 2022. 35118380 10.1093/nargab/lqac007 PMC8808542

[37] Krakau S. nf-core/mag: mag 2.5.1, November 2023.

[38] Li D. MEGAHIT: an ultra-fast single-node solution for large and complex metagenomics assembly via succinct de Bruijn graph. Bioinformatics, 31(10):1674–1676, 01 2015. 25609793 10.1093/bioinformatics/btv033

[39] Hyatt D. Prodigal: prokaryotic gene recognition and translation initiation site identification. BMC Bioinformatics, 11(1):119, 2010. 20211023 10.1186/1471-2105-11-119 PMC2848648

[40] Kieft K. VIBRANT: automated recovery, annotation and curation of microbial viruses, and evaluation of viral community function from genomic sequences. Microbiome, 8(1):90, 2020. 32522236 10.1186/s40168-020-00867-0 PMC7288430

[41] Mallick H. Multivariable association discovery in population-scale meta-omics studies. PLOS Computational Biology, 17(11):1–27, 11 2021. 10.1371/journal.pcbi.1009442 PMC8714082 34784344

[42] Steinegger M. and Söding J.. Mmseqs2 enables sensitive protein sequence searching for the analysis of massive data sets. Nature Biotechnology, 35:1026–1028, 11 2017. 10.1038/nbt.3988 29035372

[43] Hagberg A. A. . Exploring network structure, dynamics, and function using networkx. In Varoquaux G. , editors, Proceedings of the 7th Python in Science Conference, pp. 11 – 15, Pasadena, CA USA, 2008.

[44] Shannon P. Cytoscape: a software environment for integrated models of biomolecular interaction networks. Genome research, 13(11):2498–2504, 2003. 14597658 10.1101/gr.1239303 PMC403769

[45] Flamholz Z. N. Large language models improve annotation of prokaryotic viral proteins. Nature Microbiology, 9(2):537–549, Feb 2024. 10.1038/s41564-023-01584-8 PMC11311208 38287147

[46] Virtanen P. SciPy 1.0: Fundamental Algorithms for Scientific Computing in Python. Nature Methods, 17:261–272, 2020. 32015543 10.1038/s41592-019-0686-2 PMC7056644

[47] Waskom M. L.. seaborn: statistical data visualization. Journal of Open Source Software, 6(60):3021, 2021.

[48] Charlier F. Statannotations, October 2022.

[49] T. pandas development team. pandas-dev/pandas: Pandas, February 2020.

[50] Harris C. R. Array programming with NumPy. Nature, 585(7825):357–362, September 2020. 32939066 10.1038/s41586-020-2649-2 PMC7759461

