# Supplementary material for: Distinct prokaryotic gut microbiome and proviral-immune axes of pathophysiology in Sickle Cell Disease
Source: bioRxiv. 2026 Apr 15:2026.04.13.718288. Preprint. [Version 1] doi: 10.64898/2026.04.13.718288 (PMC13104962; doi:10.64898/2026.04.13.718288)
Supplement: Supplement 1 [file NIHPP2026.04.13.718288v1-supplement-1.pdf]

bioRxiv preprint doi: https://doi.org/10.64898/2026.04.13.718288; this version posted April 15, 2026. The copyright holder for this preprint (which was not certified by peer review) is the author/funder, who has granted bioRxiv a license to display the preprint in perpetuity. It is made available under aCC-BY 4.0 International license.

Supplemental

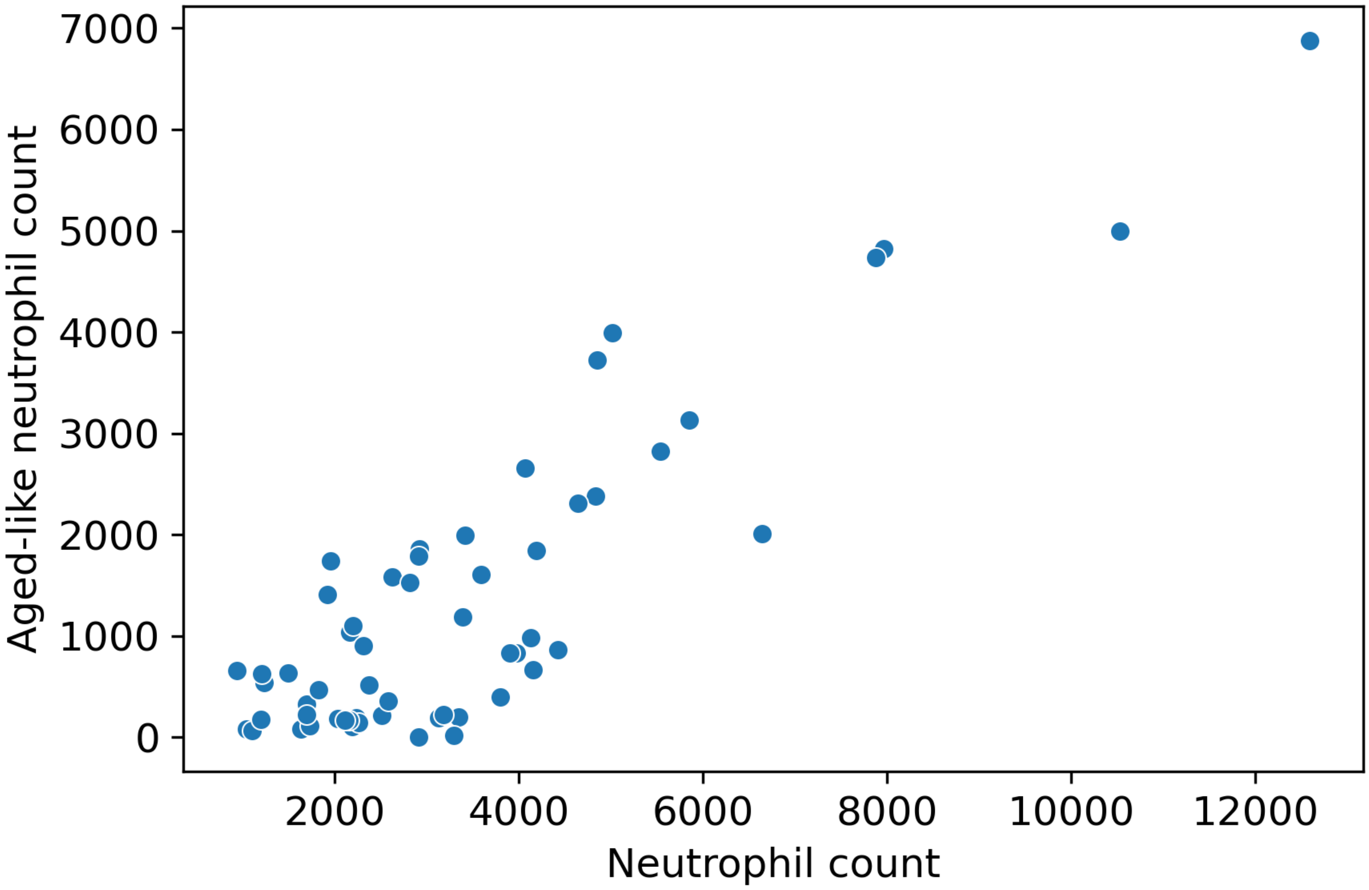

Supplemental Figure 1: SCD patient blood neutrophil profiling. Neutrophil and AN counts were assayed by flow cytometry.

bioRxiv preprint doi: https://doi.org/10.64898/2026.04.13.718288; this version posted April 15, 2026. The copyright holder for this preprint (which was not certified by peer review) is the author/funder, who has granted bioRxiv a license to display the preprint in perpetuity. It is made available under aCC-BY 4.0 International license.

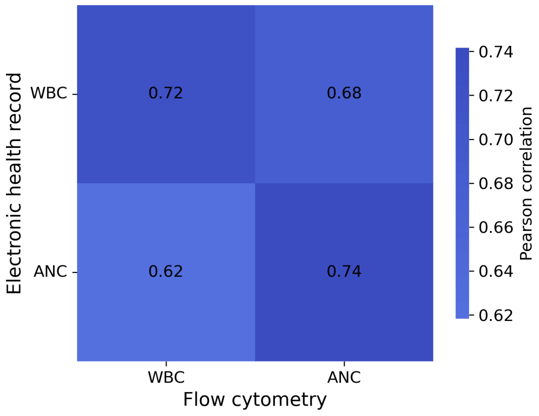

Supplemental Figure 2: Correlation of flow cytometry and EHR measures of patient white blood cell populations. Correlation measured with Pearson correlation coefficient. Abbreviations: WBC- white blood cells, ANC- absolute neutrophil count.

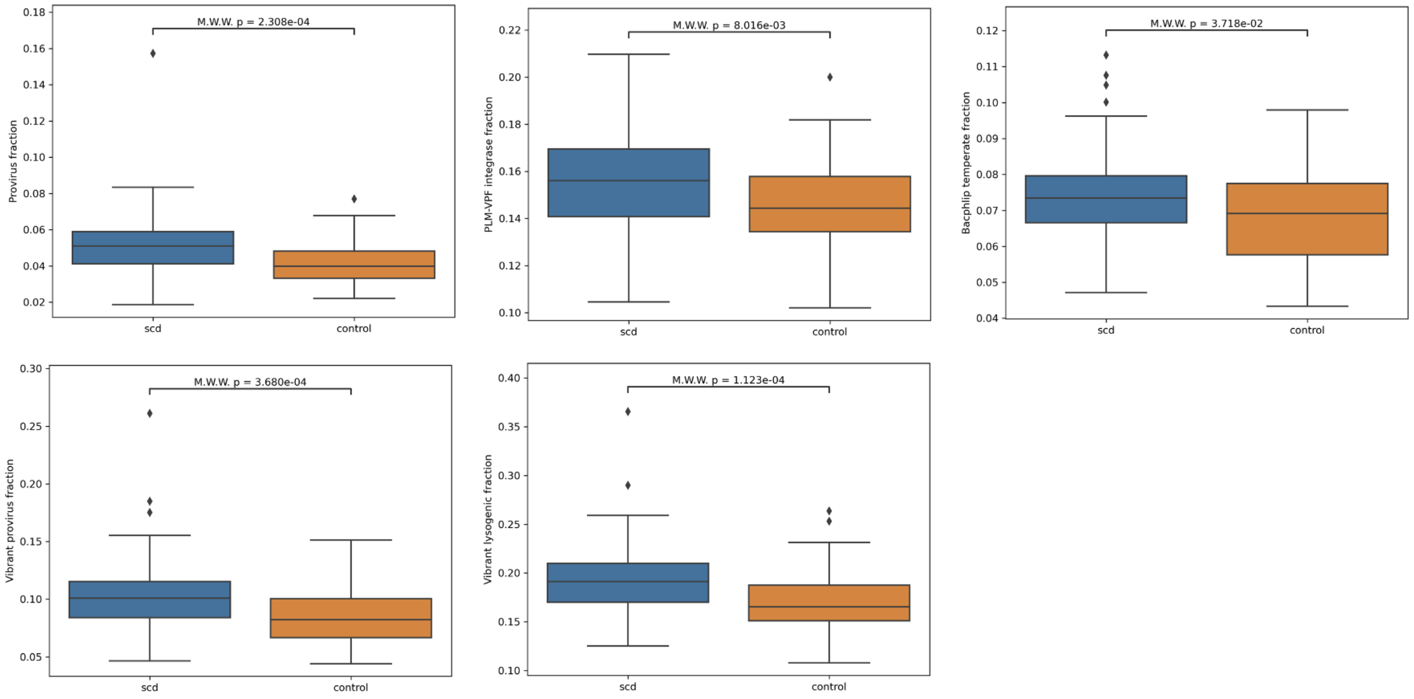

Supplemental Figure 3: Enrichment in provirus or lysogenic virus prediction across multiple labeling methods. The determination of a lysogenic virus can be made by either looking for the virus sequence integrated into a host genome or by predicting that a viral sequence has lysogenic potential. We utilized multiple methods that rely on different approaches to determine that the provirus enrichment observed is robust to method.

bioRxiv preprint doi: https://doi.org/10.64898/2026.04.13.718288; this version posted April 15, 2026. The copyright holder for this preprint (which was not certified by peer review) is the author/funder, who has granted bioRxiv a license to display the preprint in perpetuity. It is made available under aCC-BY 4.0 International license.

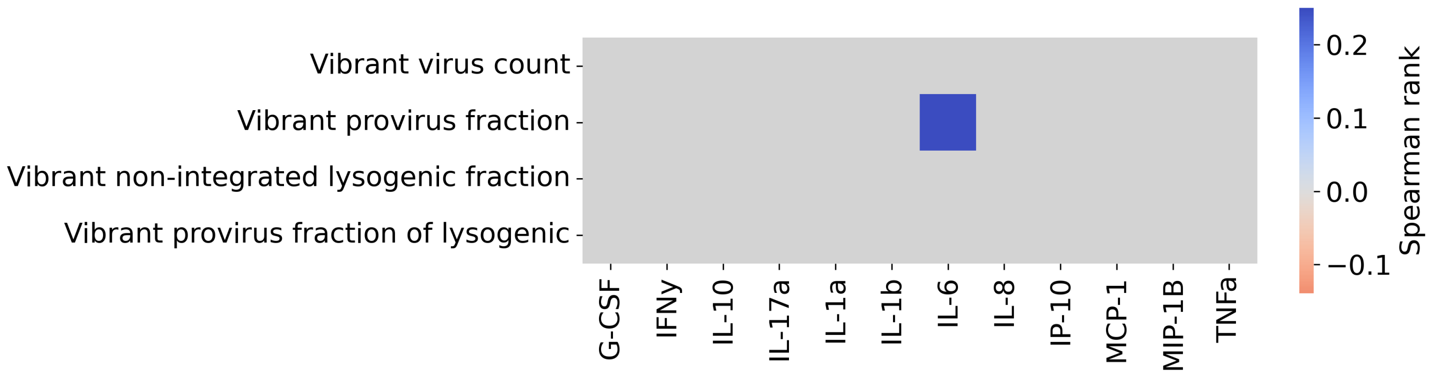

Supplemental Figure 4: Provirus fraction but not non-integrated lysogenic virus fraction correlate with immune cytokines. Using the vibrant viral calling method, the fraction of non-integrated, lysogenic predicted viruses was quantified and compared to the provirus fraction for correlation with molecular cytokines.

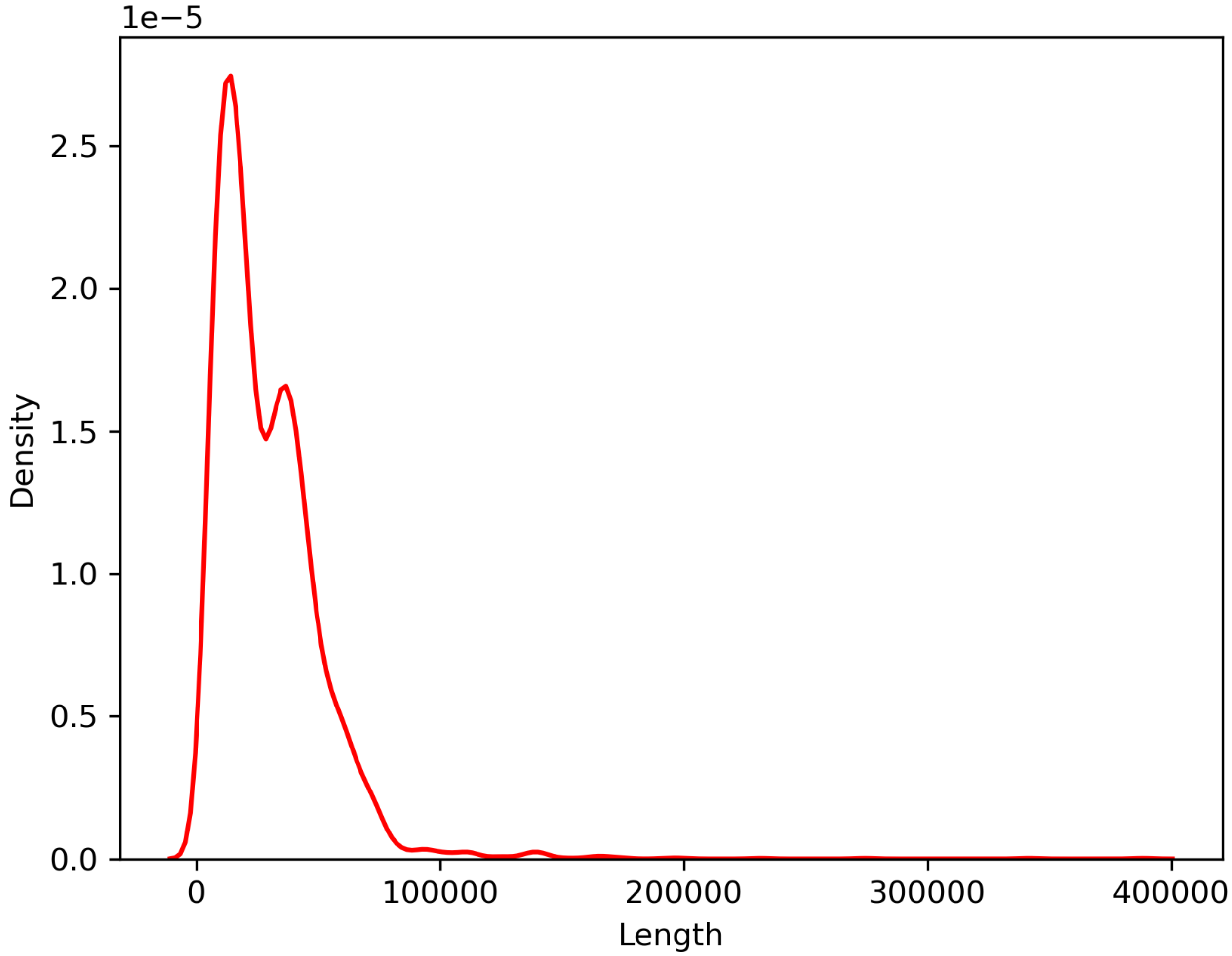

Supplemental Figure 5: Provirus length distribution with kernel density estimation.

bioRxiv preprint doi: https://doi.org/10.64898/2026.04.13.718288; this version posted April 15, 2026. The copyright holder for this preprint (which was not certified by peer review) is the author/funder, who has granted bioRxiv a license to display the preprint in perpetuity. It is made available under aCC-BY 4.0 International license.

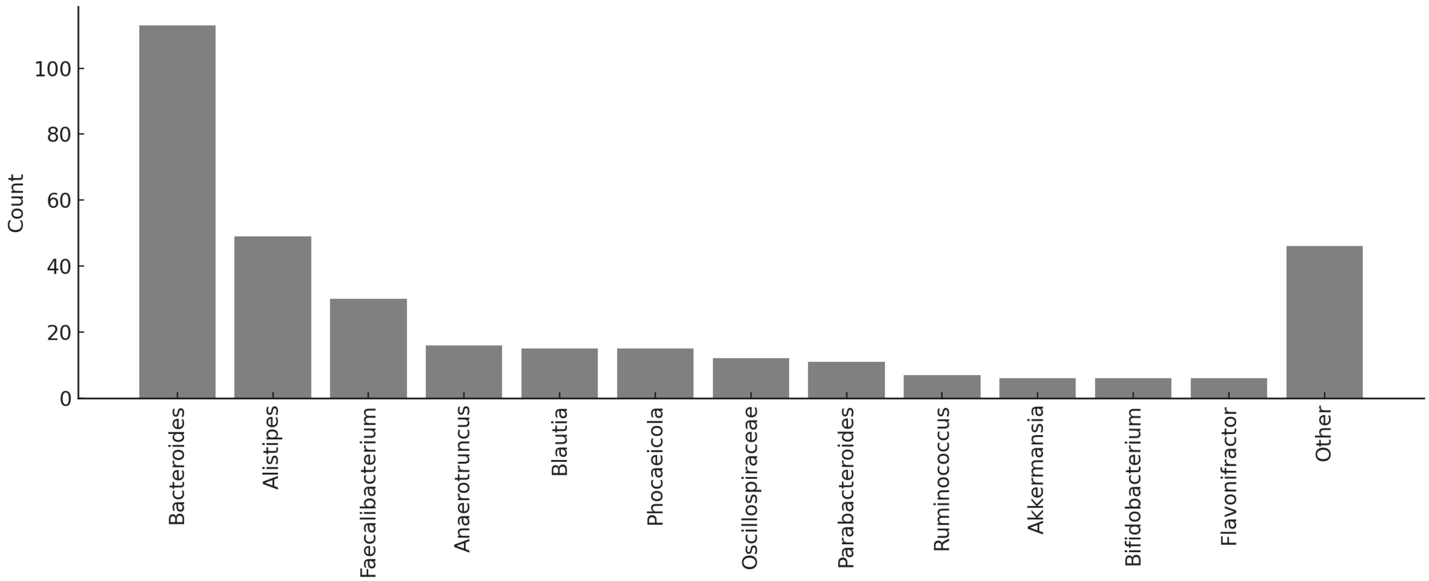

Supplemental Figure 6: Prophage cluster sequence homology with bacterial hosts. Representative cluster sequences with blast sequence homology to bacterial species sequences were aggregated to the genus level. Count represents the number of clusters homologous to species in the genus.

bioRxiv preprint doi: https://doi.org/10.64898/2026.04.13.718288; this version posted April 15, 2026. The copyright holder for this preprint (which was not certified by peer review) is the author/funder, who has granted bioRxiv a license to display the preprint in perpetuity. It is made available under aCC-BY 4.0 International license.

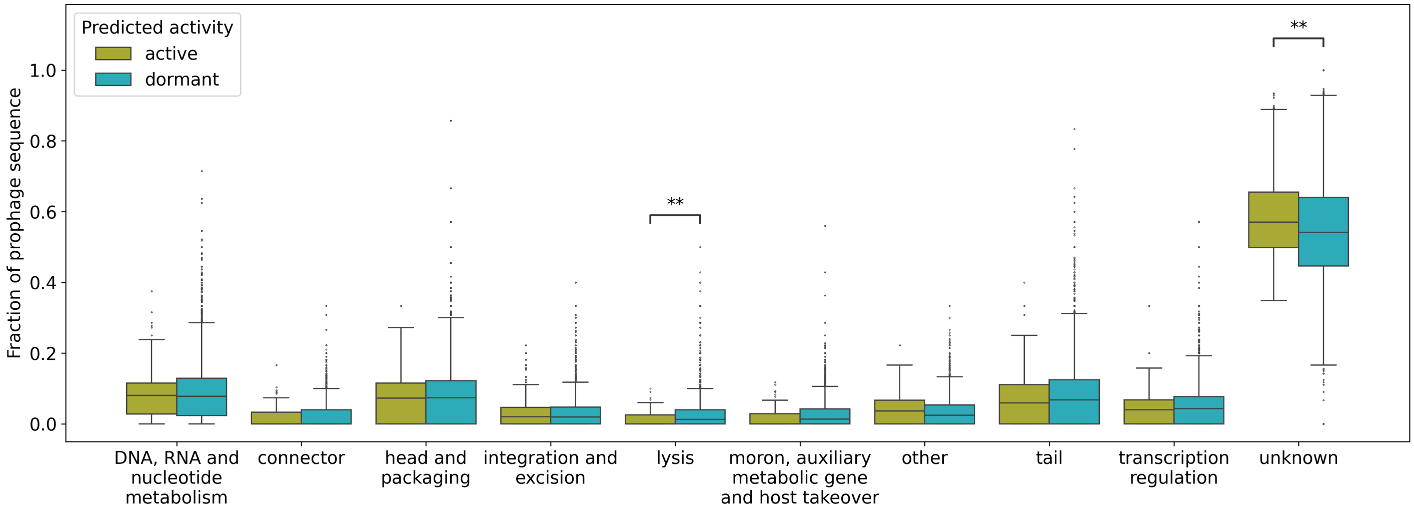

Supplemental Figure 7: Genome content comparison between predicted active (n=136) and dormant (n=4739) prophages in the SCD gut microbiome. Significance tested with a Mann-Whitney-Wilcoxon test: * = $0.01 <= p < 0.05$; ** = $0.001 < p <= 0.01$.

bioRxiv preprint doi: https://doi.org/10.64898/2026.04.13.718288; this version posted April 15, 2026. The copyright holder for this preprint (which was not certified by peer review) is the author/funder, who has granted bioRxiv a license to display the preprint in perpetuity. It is made available under aCC-BY 4.0 International license.

	SCD (n=98)	control (n=46)
Sex (Female) [%]	49	65
Ethnicity (Non-Hispanic; Hispanic; Unknown) [%]	78; 20; 2	74; 26; 0
Race (Black; White; American Indian; Multiple race; Other/Unknown) [%]	80; 1; 1; 1; 17	74; 0; 0; 0; 26
Genotype [Hb type]	SS 92%, Sβ-thalassemia 4%, SCD with high HbF 4%	AS 61%, AA 39%
Age [mean (standard deviation)]	18.2 (12.8)	18.6 (14.9)

Supplemental Table 1: Study participant demographics by cohort.

History (number with measure)	SCD patients [%]
Asthma (97)	24
Environmental allergies (97)	43
Stroke (96)	7
Acute chest syndrome (97)	6
Bacteremia (96)	26
History of bacteremia, meningitis, osteomyelitis, or urinary tract infection (96)	30
Acute care visits in past year (97)	45
ED visit in past year (97)	35
Pain admission in past year (97)	22

Supplemental Table 2: SCD patient (n=98) medical history.

Treatment (number with measure)	SCD patients [%]
HU (98)	62
Folic acid (97)	72
Glutamine (97)	16
Transfusions in past year (97)	37

Supplemental Table 3: SCD patient (n=98) treatment history.

bioRxiv preprint doi: https://doi.org/10.64898/2026.04.13.718288; this version posted April 15, 2026. The copyright holder for this preprint (which was not certified by peer review) is the author/funder, who has granted bioRxiv a license to display the preprint in perpetuity. It is made available under aCC-BY 4.0 International license.

Measure (number with measure)	Mean	Standard deviation
BMI [kg/m^2] (92)	19.9	5.0
LDH [U/L] (96)	515.4	190.4
PLT [k/uL] (97)	373.9	123.6
WBC [k/uL] (97)	9.5	3.6
ANC [k/uL] (97)	4.7	2.4
ARC [k/uL] (97)	288.3	136.4
Total bilirubin [mg/dL] (97)	3.1	2.3
Direct bilirubin [mg/dL] (95)	0.4	0.2
Indirect bilirubin [mg/dL] (97)	2.6	2.3
ALT [U/L] (97)	20.1	16.0
Creatinine [mg/dL] (97)	0.6	0.3

Supplemental Table 4: Clinical measures for SCD patients (n=98).

Measure (number with measure)	Mean	Standard deviation	Fraction above lower limit of detection
Cytokines			
G-CSF [pg/mL] (70)	18.6	31.9	36%
IFNγ [pg/mL] (85)	20.4	31.7	62%
IL-1α [pg/mL] (87)	68.4	122.9	99%
IL-1β [pg/mL] (90)	4.1	8.1	54%
IL-6 [pg/mL] (74)	20.5	33.7	99%
IL-10 [pg/mL] (95)	33.6	82.9	73%
IL-17A [pg/mL] (91)	5.1	8.5	58%
TNF-α [pg/mL] (67)	4.5	5.1	73%
Chemokines			
IL-8 [pg/mL] (94)	49.1	104.6	88%
IP-10 [pg/mL] (91)	329.5	264.8	99%
MCP-1 [pg/mL] (92)	67.5	52.4	99%
MIP-1 [pg/mL] (92)	18.2	9.9	98%

Supplemental Table 5: Molecular inflammatory measures for SCD patients (n=98).
